# Understanding patterns of temporary method use among urban women from Uttar Pradesh, India

**DOI:** 10.1186/1471-2458-14-1018

**Published:** 2014-09-29

**Authors:** Janine Barden-O’Fallon, Ilene S Speizer, Lisa M Calhoun, Livia Montana, Priya Nanda

**Affiliations:** Carolina Population Center, University of North Carolina, Chapel Hill, USA; Gillings School of Global Public Health, Department of Maternal and Child Health, University of North Carolina, Chapel Hill, USA; Harvard Center for Population and Development Studies, Harvard School of Public Health, Cambridge, MA USA; International Center for Research on Women, Asia Regional Office, New Delhi, India

**Keywords:** Contraceptives, Family planning, India, Contraceptive patterns, Reversible methods, Condoms, Traditional methods, Urban

## Abstract

**Background:**

Almost one in five contraceptive users in India uses a temporary method. It is important to understand user profiles and method use patterns for optimal program targeting.

This analysis examines differences in demographic characteristics, discontinuation and use patterns of temporary method users among a representative sample of urban women from four cities in Uttar Pradesh, India.

**Methods:**

Individual data from a panel of women aged 15–49 were collected in 2010 in Agra, Aligarh, Allahabad, and Gorakhpur and follow-up data from the same women were collected in 2012. A contraceptive calendar was used to collect month-by-month data on contraceptive use, non-use, discontinuation, reason for discontinuation, and pregnancy and birth, covering the approximately two-year period between the baseline and midterm surveys. The analysis sample is 4,023 non-sterilized women in union at baseline. A descriptive comparison is made of socio-demographic characteristics, fertility desires, discontinuation, method switching, and pregnancy outcomes. Reasons for discontinuation are assessed by the order of discontinuation.

**Results:**

There were a number of socio-demographic differences between users of temporary methods during the calendar period; by education, wealth, and caste. Notably, women who used only condoms during this time had the most education, were the least likely to be poor, and the least likely to be from a scheduled caste or tribe as compared to users of other temporary methods. Compared to the full sample of women, users of temporary methods during this period were less likely to reside in slum areas. The group of multiple method users was small in comparison to the groups of women using a single method throughout the calendar period. This indicates that there was little method switching between condoms, traditional methods, and other forms of modern methods reported in the calendar.

**Conclusions:**

The calendar may not be well-suited to measure coital-dependent contraceptive use (e.g., condoms and traditional methods), as "continuous" monthly use may be overstated. A coital episode-specific data collection tool may produce more accurate records of contraceptive use in such contexts. Research findings also lead to useful programmatic recommendations for addressing unmet need and unintended pregnancies in urban Uttar Pradesh and beyond.

## Background

Female sterilization continues to prevail as the most dominant family planning method provided and used in India. With an overall national contraceptive prevalence rate (CPR) of 56% among currently married women, the most recent National Family Health Survey (NFHS) from 2005–06 shows that female sterilization accounts for 66% percent of the share, having declined only one percentage point since 1992–93 [1]. In contrast, the share of use held by the three most common modern temporary methods (male condoms, oral contraceptive pills, and intrauterine device (IUD)) has increased slightly from 14% in 1992–93 to 18% in 2005–06 [1]. Condoms alone account for 9% of use and together with the rhythm method (9%), a traditional method, are the most popular forms of temporary method use in the country [1]. The slow increase in the prevalence of temporary method use is not trivial as the national family planning program historically placed its maximum emphasis on sterilization as a limiting method. Coercive male sterilization drives in the seventies led to the falling of the ruling government and thus stigmatized the method completely, placing the burden of the program on female sterilization [2]. In the last decade or so, the family planning program has modified its focus to increase attention to methods to delay and space births [2–4].

In general, Indian women who use temporary methods have a different socio-demographic profile than do their counterparts who use permanent methods (either female or male sterilization). Women who use temporary methods are better educated and economically better off than are women who are sterilized [1, 5]. In addition, women who use modern temporary methods are more likely to be urban than are women who are sterilized and women who use traditional methods [1]. This profile reflects the effects of targeting poor, often rural, women with sterilization campaigns, as well as the advantages of being in an urban environment with greater access to services and choice of method, especially for certain segments of the urban population. It is worth noting that the benefits of urban environments do not necessarily reach all segments of the population equitably, particularly, residents of slums, the poor, and other socially marginalized groups [5–7].

Unlike women who are sterilized, women who use temporary methods are at risk of method failure and discontinuation while in need (i.e. discontinuing contraceptive use when she wants to delay or avoid a pregnancy). As temporary method use increases, chances of method failure and discontinuation may increase. Discontinuation while in need contributes to unmet need for family planning; unmet need is the percentage of sexually active women who want to delay or avoid a pregnancy and are not using an effective method of contraception. In 2005–06, unmet need for family planning in India was at 13% [1]. Pregnancies that result from method failure and discontinuation while in need contribute to unintended pregnancy, abortion, unwanted fertility, and the overall fertility rate. More than one in five babies born in India is either mistimed (10%) or unwanted (11%), while one in seven women have had a pregnancy end in a non-live birth (e.g., spontaneous abortion, induced abortion, miscarriage, or stillbirth) [1].

In India, the one year discontinuation rate for all methods is estimated at 27%; however, discontinuation for specific temporary methods is much higher. The highest rates of discontinuation within the first year of use are for injectables (53%), pills (49%), condoms (45%), the rhythm method (32%), and withdrawal (35%) [1, 8]. While the overall discontinuation rate in India is lower than that seen in other countries due to the contribution of sterilization, the discontinuation rates for temporary methods are quite similar. For example, Demographic and Health Survey data collected between 2002 and 2009 from 19 countries in Africa, Asia, Eastern Europe and Latin America indicate a combined all country, all method discontinuation rate of 38% within the first year of use [9]. This global analysis showed that discontinuation of condoms is highest (50%), followed by pills (44%), the injectable (40%), and traditional methods (including periodic abstinence and withdrawal each at 40%) [9]. Underlying the fact that most temporary method use in India is for child spacing, the most common reason for discontinuation is to become pregnant, followed by the concern over side effects and health [1]. In other countries, typically the concern over side effects and health is the main reason for discontinuation [9].

The setting for the current study is Uttar Pradesh; a centrally located state in northern India. It is the most populous state of the country, with approximately 200 million inhabitants, 22% of whom live in urban areas [10]. The total fertility rate in Uttar Pradesh is 3.8 births per woman, as compared to the average for India, which is 2.7 (all data in this section from the NHFS-3, 2005–06) [1]. The total wanted fertility rate in the state is much lower, at 2.3, indicating that on average, women in Uttar Pradesh are having 1.5 more children than desired. The CPR for the state is 43.6%, with unmet need at 21%. As with India as a whole, sterilization is the most common method of family planning and is used by 17.3% of currently married women (accounting for about 40% of all use). Traditional methods are used by 14.3% of currently married women, condoms by 8.6%, and pills and IUD by 1.7% and 1.4%, respectively. Method discontinuation during the first year is highest for women using the pill (69.9%), condom (45.2%), or rhythm method (34.1%).

Research on the determinants of contraceptive discontinuation in Uttar Pradesh from the mid-1990’s found that the risk of discontinuation for non-method related reasons (for example, wanting to become pregnant or experiencing a change in marital status) significantly decreased with age (women ages 21–49 years versus 13–20 years), urban residence (versus rural residence), economic status (possession of a vehicle or television), travel time of less than one hour to the nearest clinic, and use of public or private sources for obtaining methods (versus commercial sources) [11]. Discontinuation for method related reasons (such as side effects, health concerns, or inconvenience) increased with a greater availability of methods and decreased with a public or private source of method. The risk of discontinuation for all reasons decreased with duration of method use [11]. Though education is often a factor associated with family planning use, it was not found to be associated with discontinuation [11]. As is currently the case, condoms were the most common temporary method used in the study from the mid-1990s [11].

### Objectives

This analysis examines differences in demographic characteristics, discontinuation and use patterns of temporary method users among a representative sample of urban women from four cities in Uttar Pradesh, India, using longitudinal data collected in 2010 and 2012. In particular, differences between women that use condoms, other temporary modern methods (pills, IUD, injectables, Standard Days Method, spermicide, emergency contraception, Lactational Amenorrhea, and "other") and traditional methods are explored. The focus is on socio-demographic characteristics of different method users, fertility desires, patterns of discontinuation and switching between methods, reasons for discontinuation, and pregnancy outcomes within a two year period.

In the coming decades, most global population growth will occur in cities, particularly migration of poor people from rural areas into urban slums. Most traditional sources of demographic survey data do not include a large enough study population to permit detailed urban analyses especially by type of residence, i.e. slum and non-slum populations. This paper adds to the evidence base on the use of temporary methods and birth spacing, specifically among condom users in urban settings of Uttar Pradesh. Like other health indicators, family planning use varies in urban settings based on wealth and place of residence (e.g., slums/non-slums) [5, 12]. This analysis also contributes to the growing body of research on urban health by considering wealth and slum/non-slum residence among the characteristics that differentiate users of different types of temporary methods.

## Methods

The data for this study come from baseline and midterm surveys conducted in urban Uttar Pradesh, India in 2010 and 2012 by the Measurement, Learning & Evaluation (MLE) Project led by the Carolina Population Center in partnership with the International Center for Research on Women. These data were collected as part of the evaluation of the Urban Health Initiative (UHI), the India arm of the Bill & Melinda Gates Foundation Urban Reproductive Health Initiative. Individual data from a panel of women aged 15–49 in 2010 were collected in Agra, Aligarh, Allahabad, and Gorakhpur and follow-up data from the same women were collected in 2012. These cities were included based on selection by the UHI program as the initial intervention cities and are not meant to represent urban Uttar Pradesh overall. The midterm data were collected from about half of the baseline sample of women; details have been described previously [13]. Half the sampling units were located in slum areas and half were located in non-slum areas. Slum areas were purposely oversampled to allow for stratified slum/non-slum analysis; however, to adjust the sample so that it is representative of the cities, sample weights are used to assess socio-demographic characteristics. All study procedures were approved by the Institutional Review Boards of the Futures Group in India and by the University of North Carolina at Chapel Hill.

The questionnaires collected information on common socio-demographic characteristics, such as age, education, parity, and fertility desires. At midterm, a contraceptive calendar was used to collect month-by-month data on contraceptive use, non-use, discontinuation, reasons for discontinuation, and pregnancy and birth experience, covering the approximately two-year period between the baseline and midterm surveys. The calendar started in January 2010 and covered the period until the date of the midterm interview, which fell between March – May 2012. Up to four columns of information were collected for each month during the period. The columns were filled in through a series of questions embedded throughout the survey. The first column indicated the marital status of the respondent. The second column collected mutually exclusive indicators on whether the respondent was either pregnant, gave birth, experienced a pregnancy termination, or was using/not using a contraceptive method in each month. The third column contained a code for the source from which a new contraceptive method was obtained during the first month of the episode of new use. Finally, the last column provided a code for the reason for discontinuation of the method recorded in the last month that the method was used for each episode of use in the calendar.

There were a total of 5,790 women who provided information to the contraceptive calendar. Women who reported using female sterilization or male sterilization in the first month of the calendar (1,135) are included in Table 1 for comparison only. Calendar information was not collected for women reporting a hysterectomy or being in menopause at the time of the mid-term survey, and these women (n = 632) are removed from further analysis. Thus, the analysis sample is 4,023 non-sterilized, fecund women in union at baseline. The calendar includes information on contraceptive use for a minimum of 26 months and a maximum of 28 months for each of these women.

**Table 1:** **Characteristics and fertility intentions of sampled women living in four cities in urban Uttar Pradesh, India, 2010 and 2012**

Characteristic*	Full sample @ mid-term N = 5,790** (100%)	Sterilized @ start of calendar n = 1,135 (19.6%)	No use during calendar n = 1,406 (24.3%)	Temporary method use during calendar				Sterilized during calendar n = 137 (2.4%)
				Condom n = 948 (16.4%)	Traditional n = 977 (16.9%)	Other modern^ n = 407 (7.0%)	Multiple reversible methods^^ n = 148 (2.6%)	
**Mean age (years)**	36.1	39.1	33.9	32.5	34.5	33.1	31.1	31.5
**Education**								
None	28.9	37.2	34.1	12.2	25.9	19.2	24.5	36.1
1-11 years	36.5	43.1	35.9	34.0	35.7	33.4	38.1	36.2
12+ years	34.6	19.7	30.0	53.8	38.4	47.4	37.4	27.7
**Mean parity (# live births)**	3.0	2.9	3.1	3.1	2.9	3.0	3.3	2.9
**Household wealth**								
Poorest	17.3	19.0	25.0	10.2	14.8	16.0	16.7	24.7
Poor	19.8	21.5	21.9	14.6	21.2	20.5	21.7	27.0
Medium	20.6	21.9	18.5	19.6	21.8	15.4	25.3	20.1
Rich	20.6	17.4	18.9	27.7	21.5	23.1	14.4	13.7
Richest	21.7	20.2	15.9	28.0	20.8	24.9	21.9	14.5
**Caste (Baseline)**								
Scheduled caste or tribe	22.1	26.3	23.9	16.1	23.6	16.1	21.3	30.3
Other BW caste	40.5	32.7	41.1	36.3	34.5	46.0	42.3	41.7
DK/None of above	40.0	41.0	35.0	47.6	41.9	37.9	36.4	28.0
**City**								
Agra	34.1	32.2	34.1	32.9	37.8	32.1	24.5	45.9
Aligarh	16.7	8.9	23.3	19.9	16.2	19.6	21.2	10.9
Allahabad	28.5	34.2	22.9	29.0	26.5	26.1	36.1	21.8
Gorakhpur	20.7	24.8	19.5	18.2	19.5	22.1	18.1	21.4
**Residence**								
Slum	16.2	16.7	19.9	14.0	14.7	13.6	13.1	22.9
Non-slum	83.8	83.3	80.1	86.0	85.3	86.4	86.9	77.1
**Fertility intentions (Baseline)**								
Wants now, within 2 yrs	8.6	0.1	23.0	9.3	8.0	4.2	4.7	9.5
Wants 2+ yrs	12.5	0.1	19.6	19.8	18.3	14.2	13.5	8.9
Wants no more	45.7	3.9	46.9	69.7	70.9	79.0	78.4	62.4
Can’t get pregnant/ DK	33.2	96.0	10.5	1.2	2.8	2.5	3.3	19.2

*Assessed at mid-term and presented as % distribution unless otherwise noted.

**632 women (10.9% of full sample) who were in menopause or had a hysterectomy at the time of the mid-term survey did not contribute information to the calendar and are dropped from further analysis.

^Other modern methods include oral contraceptive pills, IUD, injectables, "other", Standard Days, spermicide, emergency contraception and Lactational Amenorrhea.

^^Any combination of condom, traditional methods, or other modern temporary methods used during the calendar.

Patterns of contraceptive use in the calendar are assessed to construct meaningful categories of women’s contraceptive use. These categories allow for a descriptive comparison of socio-demographic characteristics, fertility desires, discontinuation and method switching, and pregnancy outcomes within a two year period. Reasons for discontinuation are assessed by the order of the discontinuation (whether 1^st^, 2^nd^ or 3^rd^) to better understand how motivations for discontinuation vary by a first or higher order discontinuation. All data presented use midterm weights that adjust for non-response and sampling design.

## Results

Over the course of the calendar, a total of 2,617 women used a method for at least one month. A comparison of women’s characteristics by contraceptive method use is shown in Table 1. Women were categorized as: sterilized at the start of the calendar (19.6% of full sample); no method use during the calendar (24.3%); condom use only during the calendar (16.4%); traditional method use (periodic abstinence/rhythm or withdrawal) only during the calendar (16.9%); other modern method use^a^ (mainly pills, IUD, injectables) only during the calendar (7.0%); use of more than one temporary method (any combination of condoms, traditional, or the other various modern methods) during the calendar (i.e. "multiple") (2.6%); or whether they had been sterilized during the calendar period (2.4%). A woman qualified as a method user during this period with as little as one month of use. The categories are mutually exclusive; method switching is captured from women who used multiple methods or used a method and were later sterilized during the calendar period. Women using multiple methods most commonly used condoms and other modern methods (n = 65); few women switched between condoms and traditional methods (n = 13) or between all three types of temporary methods (condoms, traditional and other modern methods) (n = 8). Of the 137 women who were sterilized during the calendar period, 33 (24%) had used another method of contraception during the calendar period; the remaining did not use any other method during the calendar period prior to sterilization.

As seen in Table 1, there are some differences between women who were using temporary methods and those that were sterilized at the beginning of the calendar period. For example, women who were sterilized at baseline were, on average, older, less educated, more likely to come from the poorest households, and more likely to reside in slum areas, than were women who used temporary methods during the calendar period. Non-users were similar to women who had become sterilized during the calendar period (many of whom had been non-users prior to the sterilization), and were less educated, more likely to be from the poorest households, and more likely to reside in slum areas than women using temporary methods. While non-users were the most likely at baseline to report wanting additional children "now" or within two years, this was the expressed desire for only 23% of nonusers; a full two-thirds of non-users expressed a desire for spacing or limiting births.

Likewise, there are differences between the categories of temporary method users. A greater percentage of women who only used condoms during the calendar period, for example, have 12+ years of education as compared to other groups of temporary method users. A lower percentage of condom users are from the poorest households or from a scheduled caste or tribe, while a greater percentage report a desire to delay the next birth for two or more years. Overall, there is not much difference in slum residence among users of temporary methods. Finally, the percentage of women who desired no more children at the baseline interview was fairly consistent across temporary method users (69.7-79.0%) while less than 10% of temporary method users desired a child within the next two years.

Table 2 shows a comparison of method use characteristics using the same categories of method users as depicted in Table 1. The mean number of months of method use was highest for women using traditional methods (23.3 months) and condoms (22.9 months) during the calendar period. The number of discontinuations recorded in the calendar was small. A total of 522 women discontinued a method at least once during the period (20% of temporary method users); though most of these women discontinued only once (90%), there were as many as 6 discontinuations per woman during the period (1 woman). The (weighted) proportion of women who discontinued at least once during the two-year calendar period was 0.137 (13.7%; unweighted n = 522); of these only 0.122 (12.2%; unweighted n = 54) discontinued more than once. A total of 1,014 women initiated a method at least once during the calendar (25% of women with calendar data). The mean number of discontinuations and initiations per woman was highest among multiple method users and women getting sterilized during the calendar, as would be expected given that these categories include method switching. These two groups of women were also the most likely to spend at least one month as a non-user during the calendar period (including non-use due to pregnancy, birth or termination). This indicates that method switching was not happening in consecutive months. Interestingly, the mean number of pregnancies and terminations during the calendar period was lowest for women using temporary modern methods other than the condom. Pregnancies were most common for women who then got sterilized and for women using multiple methods followed by non-users. Additionally, terminations were the most common for women using multiple methods; in fact, terminations were more than three times higher for women in this group than for women who had no method use during the calendar period and more than thirteen times higher than for women who used a temporary modern method other than condoms, though the numbers are very small.

**Table 2:** **Method use characteristics of sampled women living in four cities in urban Uttar Pradesh, India, 2010 and 2012**

Characteristic	Full Sample @ mid-term N = 5,790 (100%)	Sterilized @ start of calendar n = 1,135 (19.6%)	No use during calendar n = 1,406 (24.3%)	Temporary method use during calendar				Sterilized during calendar n = 137 (2.4%)
				Condom n = 948 (16.4%)	Traditional n = 977 (16.9%)	Other modern* n = 407 (7.0%)	Multiple reversible methods** n = 148 (2.6%)	
**Mean # months of use during calendar**	NA	NA	0	22.9	23.3	21.0	21.9	13.2^
**Mean # discontinuations during calendar**	NA	NA	0	0.16	0.14	0.16	1.36	0.31
**% women that had at least 1 month non-method use**	NA	NA	100	36.6	33.9	44.0	55.2	77.5
**Mean # initiations from non-use during calendar**	NA	NA	0	0.31	0.27	0.37	1.50	1.03
**Mean # pregnancies during calendar**	NA	NA	0.49	0.36	0.27	0.26	0.59	0.62
**Mean # terminations during calendar**	NA	NA	0.08	0.08	0.04	0.02	0.27	0.05

*Other modern methods include oral contraceptive pills, IUD, injectables, "other", Standard Days, spermicide, emergency contraception and Lactational Amenorrhea.

**Any combination of condom, traditional methods, or other modern temporary methods used during the calendar.

^The mean number of months of use of sterilization only.

NA – not applicable for this group of women.

A closer examination of use indicates that 683 women (17%) became users during the course of the calendar period and did not discontinue by the time of the mid-term survey (results not shown in tables). However, the majority of women (70%) did not change status during the calendar period- either remaining a non-user throughout the calendar (35%) or remaining a user of the same method throughout the calendar (35%). The calendar data also reveal that there were 109 women who used a contraceptive during the calendar period (most often traditional methods (49%), but also condoms (31%), other (18%), and multiple methods (2%)), who were non-users at the time of the baseline and mid-term surveys. This means that 11% of non-users (109/980) recorded at the two points in time had actually used contraception at least one month during the period between surveys.

Table 3 presents the reasons for discontinuation reported by the 522 women who discontinued at least once during the calendar. Discontinuations of the first, second, and third order are included in the table, whereas discontinuations of the 4^th^, 5^th^, and 6^th^, order are not as they accounted for only six discontinuations in total (each of the six higher order discontinuations was due to problems with the method). The weighted percent of women reporting each reason is shown in the table by whether it was a first, second, or third order discontinuation.

**Table 3:** **Main reason for discontinuation, by order of discontinuation, from sampled women living in four cities of urban Uttar Pradesh, India, 2012 (N = 522)**

Reason for discontinuation	1 ^st^ n = 522	2 ^nd^ n = 54	3 ^rd^ n = 9	Total n = 585
**Problems with method (%)**	**47.0**	**63.9**	**74.6**	**47.6**
Became pregnant while using	18.7	14.8	36.6	17.5
Wanted more effective method	13.6	12.9	1.8	13.6
Health concerns	6.6	22.1	31.2	7.5
Side effects	6.7	9.5	5.0	7.1
Inconvenient to use	1.4	4.6	0.0	1.9
**Diminished demand (%)**	**43.9**	**33.5**	**1.4**	**43.6**
Wanted to become pregnant	31.7	20.2	1.4	31.4
Difficult to get pregnant/menopausal	8.0	0.0	0.0	8.0
Infrequent sex/husband away	4.2	13.3	0.0	4.2
**Other (%)**	**9.2**	**2.6**	**23.8**	**8.8**
Husband/partner disapproved	2.9	2.3	23.8	2.5
Lack of access/too far	0.2	0.0	0.0	0.2
Other	6.1	0.3	0.0	6.1

As seen in Table 3, the most commonly reported reasons for a first, second or third discontinuation are wanting to become pregnant (31.4%), method failure (or becoming pregnant while using the method) (17.5%), and wanting a more effective method (13.6%). The pattern is a little different when comparing first discontinuations against second and third; while first discontinuations are most commonly due to a desire to become pregnant (31.7%), second discontinuations are most likely due to health concerns (22.1%) and third discontinuations are most commonly due to method failure (36.6%), though the number of third discontinuations is very small.

After grouping together reasons for discontinuation as "problems with method", "diminished demand" and "other", reasons related to problems with the method are the most common (47.6%) reasons for first discontinuation, followed closely by diminished demand for contraception (43.6%), and lastly, other reasons related to supply, partner disapproval and "other" (8.8%). If we look at 2^nd^ and 3^rd^ order discontinuations (n = 63) however, problems with method account for about two-thirds of the discontinuations.

A further analysis of discontinuation behavior shows that the majority of discontinuations were followed by non-use (46.8%) or pregnancy (25.6%) in the month following the discontinuation. Discontinuations were next most likely to be followed by use of reversible modern methods other than the condom (13.0%). Discontinuations were least likely to be followed by initiations of condoms (6.0%), sterilization (4.8%), or traditional methods (3.8%) in the month following discontinuation. Method use following discontinuation is presented in Table 4, by method used at the time of discontinuation and by order of discontinuation.

**Table 4:** **Method use during the month following discontinuation, by order of method discontinuation, from sampled women living in four cities of urban Uttar Pradesh, India, 2012 (N = 522)**

	Method used (%)						
Method discontinued (n)	Non-use	Pregnant	Condom	Traditional	Other modern	Sterilization	Total
	**After 1** ^**st**^ **discontinuation (n = 522)**						
Condom (186)	50.0	30.6	1.6	1.1	11.8	4.8	100.0
Traditional (188)	47.9	34.0	2.7	3.2	5.9	6.4	100.0
Other Modern (148)	50.0	11.5	12.8	4.7	16.9	4.1	100.0
	**After 2** ^**nd**^ **discontinuation (n = 54)**						
Condom (16)	25.0	31.3	0.0	6.3	37.4	0.0	100.0
Traditional (15)	33.3	26.7	13.3	0.0	26.6	0.0	100.0
Other Modern (23)	4.5	30.4	17.4	17.4	26.1	4.3	100.0
	**After 3** ^**rd**^ **discontinuation (n = 9)**						
Condom (5)	0	20.0	0.0	40.0	40.0	0.0	100.0
Traditional (1)	0	100.0	0.0	0.0	0.0	0.0	100.0
Other Modern (3)	33.3	0.0	66.6	0.0	0.0	0.0	100.0

## Discussion

This analysis examined differences in demographic characteristics and use patterns among temporary method users among a representative sample of urban women from four cities of Uttar Pradesh, India interviewed at two year intervals (2010 and 2012). Differences between women that used condoms, other temporary modern methods (i.e. pills, IUD, injectables, etc.), and traditional methods were explored. We found a number of socio-demographic differences between users of temporary methods during a 26–28 month calendar period, by education, wealth, and caste. Notably, women who used only condoms during this time had the most education, were the least likely to be poor, and the least likely to be from a scheduled caste or tribe. Compared to the full sample of women, users of temporary methods during this period were less likely to reside in slum areas. In contrast, women who became sterilized during the course of the calendar period were the most likely to be living in slum areas (22.9% compared to 16.2% of the total surveyed population). These findings are similar to those found examining use patterns at baseline in the same sample; poor and less educated women were more likely to be sterilized than richer and more educated women who were more likely to be condom and other modern method users [5].

The group of multiple method users was small in comparison to the groups of women using a single method throughout the calendar period. This indicates that there was little method switching occurring between condoms, traditional methods, and other forms of modern methods. In fact, only 6% of women who used temporary methods during the calendar period used more than one temporary method. However, the higher incidence of pregnancy and termination for users of multiple temporary methods during the two year period may be indicative of problems with switching and coverage lapses between methods. Almost all women in the group of multiple method users reported wanting to space or limit childbearing at baseline. Furthermore, compared to women who used a single method during the calendar period, women using multiple methods were most likely to report that their discontinuations were due to wanting a more effective method; these women were the most likely to experience a pregnancy during the calendar period.

Interestingly, traditional method users were among the group of temporary method users with the lowest occurrence of pregnancy and terminations (second only to women who consistently used modern methods other than the condom). Traditional method users and users of other modern methods (mainly the pill, injectables and IUD) also had pregnancy and termination rates that were lower than women who reported condom use in each monthly period. This suggests that women using condoms do not use them consistently for every sexual encounter, thus undermining the effectiveness of the method. Additionally, we see that users of condoms and multiple methods are no less likely to have pregnancy termination than non-users.

An analysis of the 1992–3 National Family Health Survey (NFHS) found that 48% of former contraceptive users in Uttar Pradesh had discontinued using contraception due to a method problem or method failure, 27% discontinued because they wanted another child, and 25% discontinued for ‘other’ reasons [14]. In our analysis of users in the calendar period, we found a similar high percentage of method discontinuation due to problems with the method, particularly for women with more than one discontinuation during the calendar period. Discontinuation related to having another child was most pronounced for women with only one discontinuation during the two year period. Discontinuation as an indicator of program quality has long been recognized in the literature [9, 11, 15, 16]. Mishra, Retherford, Nair and Feeney (1999), authors of the 1992–3 NFHS study, suggest that with improvements in family planning service quality, the percentage of women citing method problems or failure could be reduced. However, more than two decades later, method problems and failure remain among the most commonly stated reasons for discontinuation found in our sample from urban areas of Uttar Pradesh.

This study is not without limitations. First, we used the entire calendar for this analysis; as a result pregnancy estimates could be underestimated due to early pregnancies that were not yet detected by women in the final months of the calendar. Additionally, pregnancy terminations may have been underreported due to stigma associated with induced abortions, however, given that abortion is legal in India, this may not be an important source of bias.

Second, because the calendar collects contraceptive use data retrospectively, recall bias can impact reports of use occurring many months or years before the survey. In an examination of agreement between current contraceptive use status recorded in the baseline interview and what was collected in the corresponding month of the calendar, we found no higher than 66% agreement. This suggests that recall bias was indeed a factor for reporting of contraceptive behavior early in the calendar.

Another limitation of this analysis is the possibility of over-reporting in the calendar, as an abundance of women appears to have used a single method for every month of the calendar. This could overestimate the mean number of months of use and underestimate the mean number of discontinuations and initiations. This leads us to question the appropriateness of using the contraceptive calendar in a context such as this, where temporary use is dominated by coital-dependent methods of contraception such as condoms and traditional methods. The calendar, which captures method use on a monthly basis, may be a measure more suited for temporary methods that are not as commonly used in Uttar Pradesh, such as injectables, implants, pills, or the IUD. In the calendar, women using coital-dependent methods such as traditional methods and condoms would be counted as having used the method for a full month even if the method was used for a single coital episode at any time during the month, regardless of whether the method was used for any other coital episodes or used consistently. Thus what we consider ‘continuous method use’ month to month for methods such as condoms and traditional methods may in fact not be consistent use, and thus may misrepresent actual use patterns. This would also likely result in higher misreporting of past months of use. Any dual method use between condoms and traditional methods would also not be captured by the calendar. To better understand the nature of use for methods that are coital-dependent, more detailed information is necessary, perhaps through a diary or some other type of coital episode-specific tool. This has implications for the measurement and evaluation of temporary method use for India as a whole, as well as other countries with high use of coital-dependent methods.

## Conclusions

Beyond this methodological recommendation, this study also leads to useful programmatic recommendations for addressing unmet need and unintended pregnancies in urban Uttar Pradesh and beyond. In particular, we found that multiple method users are at the highest risk of discontinuation, particularly for reasons of wanting a more effective method. This is of programmatic concern, especially for the cities of Allahabad, which had 28.5% of the surveyed population but 36.1% of multiple method users, and Aligarh, which had 16.7% of the surveyed population but 21.2% of multiple method users. Improved access to a full array of methods and knowledge of the methods’ true side effects is needed to ensure that women select a method best suited to their needs. All women should understand the concept of method switching; how to change methods effectively and avoid unwanted pregnancy and what other temporary and permanent methods are available to use. This information needs to reach all women, including women who may not be coming into a clinic for their family planning methods. Finally, given that a high percentage of discontinuations were due to method related reasons, there is a need to address women’s health concerns and fear of side effects to support effective method switching to avoid method failure and subsequent unintended pregnancies. It is these types of targeted efforts that will lead to more effective use of temporary methods and in turn result in less unintended pregnancy and recourse to abortion in Uttar Pradesh, India and beyond.

### Endnote

^a^Methods in this category include all other temporary methods mentioned by women, here noted with the number of women using each method in parentheses: oral contraceptive pill (179); IUD (125); injectables (44); "other" (25); Standard Days (18); spermicide (8); emergency contraception (6); and Lactational Amenorrhea (2).

## Abbreviations

CPR: Contraceptive prevalence rate
IUD: Intrauterine device
MLE: Measurement, Learning and Evaluation Project
NFHS: National Family Health Survey.
